# Influence of therapeutic play on salivary cortisol levels in children undergoing venipuncture

**DOI:** 10.1590/0034-7167-2024-0636

**Published:** 2025-12-12

**Authors:** Carina Ceribelli, Fabiane de Amorin Almeida, Circéa Amália Ribeiro

**Affiliations:** I Universidade Cruzeiro do Sul. São Paulo, São Paulo, Brazil; II Faculdade Israelita de Ciências da Saúde Albert Einstein. São Paulo, São Paulo, Brazil; III Universidade Federal de São Paulo. São Paulo, São Paulo, Brazil

**Keywords:** Play and Playthings, Catheterization, Peripheral, Hydrocortisone, Pediatric Nursing, Hospitalization., Juegos y Juguetes, Cateterismo Periférico, Hidrocortisona, Enfermería Pediátrica, Hospitalización.

## Abstract

**Objectives:**

to investigate the influence of Instructional Therapeutic Play on salivary cortisol levels in children undergoing peripheral venipuncture.

**Methods:**

randomized controlled clinical trial, allocated into two groups: Intervention and Control, conducted in pediatric inpatient units, children’s emergency rooms, and outpatient clinics of two hospitals in the city of São Paulo. A sample of 52 children, both genders, aged between 3 and 12 years, underwent venipuncture and were able to play. Data were analyzed using descriptive and inferential statistics with a 5% significance level and a 95% confidence interval.

**Results:**

cortisol levels decreased in both groups, but less so among children who did not receive the toy intervention, remaining lower for those who did receive the intervention, and among those who were successfully punctured on the first attempt.

**Conclusions:**

although the null hypothesis was confirmed in this study, the use of Therapeutic Play for stress relief is recommended.

## RESUMEN

**Objetivos:**

investigar la influencia del Juguete Terapéutico Instructivo sobre los niveles del cortisol salivar de niños sometidos a venopunción periférica.

**Métodos:**

ensayo clínico controlado y aleatorizado, distribuido en dos grupos: Intervención y Control, realizado en unidades de hospitalización pediátrica, urgencias infantiles y ambulatorios de dos hospitales de São Paulo. Constituyeron la muestra 52 niños de ambos sexos, de 3 a 12 años, sometidos a venopunción y aptas para jugar. Los datos se analizaron mediante estadística descriptiva e inferencial, con nivel de significación del 5% e intervalo de confianza del 95%.

**Resultados:**

los niveles de cortisol disminuyeron en ambos grupos, aunque menos entre aquellos niños que no recibieron la intervención del juguete, permaneciendo más bajos para los que sí recibieron la intervención, y entre los que fueron punzados con éxito en el primer intento.

**Conclusiones:**

a pesar de confirmada la hipótesis nula en este estudio, se recomienda el uso de Juguetes Terapéuticos para aliviar el estrés.

## INTRODUCTION

The motivation for this research arose from our interest in utilizing and deepening knowledge about available care technologies to promote humanization and alleviate the suffering of children who are ill or undergoing painful and invasive procedures.

It is known that every child needs to play to communicate, express their feelings and anxieties, and understand certain situations. Play is a child’s work, an activity essential to their mental, emotional, and social well-being that remains a developmental necessity, even when the child is ill^(1)^. Furthermore, play is a right guaranteed by the Child and Adolescent Statute (CAS), which, in Article 16, establishes the child’s right to freedom, which includes playing, playing sports, and having fun^(2)^.

Playful activities, whether recreational or therapeutic, can help reduce stress and distress in children. Among these, Therapeutic Play (TP) stands out. It is traditionally conceptualized as a structured toy designed for children to alleviate anxiety caused by atypical experiences for their age, which are often threatening and require more than just recreation to resolve anxiety^(3)^.

Among the types of TP, we highlight Instructional Therapeutic Play (ITP), which should be used to demonstrate procedures, providing clear, precise, and developmentally appropriate information. It allows children to release tension by role-playing the situations they experience and handling the instruments or toys that represent them^(4)^.

Studies indicate that its use favors the reduction of behaviors indicative of stress, including during invasive procedures in healthcare, especially venipuncture^(5)^. However, no studies related to the influence of ITP on a physiological marker of stress have been identified.

Stress levels in childhood can be measured by measuring the hormone cortisol after a stressful stimulus. Cortisol is a corticosteroid hormone produced by the upper adrenal gland and directly involved in the response to stressful stimuli^(6)^. Salivary testing is recommended for children because it is non-invasive, safe, painless, and easy to perform^(7)^.

A study analyzing pain and free cortisol in newborns concluded that repeated venipuncture intensified pain and altered cortisol levels^(8)^. In another study, conducted with children aged 5 to 7 years old hospitalized for acute or chronic illnesses, morning and afternoon salivary cortisol levels were measured. A co-oping scale was used to assess stress. The study found that afternoon cortisol increased with younger children. Among the co-oping strategies for coping with stress resulting from hospitalization, play was the most frequently used by children^(9)^.

There is a lack of studies related to the effectiveness of TP in reducing salivary cortisol levels in children exposed to painful and intrusive procedures, such as venipuncture, which was the objective of this study.

Therefore, in this study, in which we used ITP as a strategy to prepare children for venipuncture, we considered the following research hypotheses to be investigated: - Alternative hypothesis (H1): The use of ITP in preparing children for venipuncture alters salivary cortisol levels; - Null hypothesis (H0): The use of ITP in preparing children for venipuncture does not alter salivary cortisol levels.

## OBJECTIVES

To investigate the influence of ITP on salivary cortisol levels in children undergoing peripheral venipuncture.

## METHODS

### Ethical aspects

In compliance with Resolution 466/12 of the National Health Council^(10)^, this study was approved by the research ethics committee of the Federal University of São Paulo - UNIFESP via Plataforma Brasil, as per the report attached to this submission. The research was also authorized by the institutions where the data were collected. Parents or guardians signed the Informed Consent Form, authorizing the children’s participation in the research, and the children signed the Assent Form.

The study was registered with the Brazilian Clinical Trials Registry (ReBEC) platform, submission code U1111-1287-3284.

### Study design, period and location

This is a randomized, controlled, parallel-group clinical trial with a 1:1 allocation, guided by the CONSORT tool. A comparative study of salivary cortisol levels was conducted to determine whether there was a reduction in salivary cortisol levels with the use of ITP, as an indicator of reduced stress in children.

Data collection took place between September 2018 and October 2022, considering a one-and-a-half-year interval due to restrictions imposed by the COVID-19 pandemic.

The study was conducted at three locations in the city of São Paulo: a pediatric hospital affiliated with the State Department of Health, which provides services in various pediatric specialties and is a referral center for the treatment of mediumand high-complexity chronic diseases; a large, high-complexity public tertiary hospital, which offers outpatient, inpatient, intensive care, surgery, and emergency care to children and adolescents across various specialties; a specialized outpatient clinic, affiliated with the same hospital, that primarily treats patients with hematologic disorders within the Unified Health System (SUS).

### Population or sample

Fifty-two children treated at these services participated in the study, 24 in the Control Group and 28 in the Intervention Group. They met the following inclusion criteria: being between 3 and 12 years of age; undergoing peripheral venipuncture during hospitalization or outpatient care; and being in a favorable clinical condition that allowed them to play. The exclusion criteria were: fasting for more than 12 hours, experiencing pain, and using glucocorticoids; sleep deprivation for more than 12 hours; and having neurological impairment or medical diagnoses related to the hypothalamic-pituitary-adrenal axis.

The dependent variable of the study was salivary cortisol, with saliva samples being collected and measured, which can be used to monitor the concentration of a wide variety of drugs, hormones, antibodies, and other molecules^(11)^. The independent variable was the ITP, the action that could affect the dependent variable. According to the literature, the dependent variable is the factor to be manipulated by the researcher in an attempt to influence and ensure its relationship with the dependent variable, in this case, salivary cortisol^(11)^.

### Study protocol

The ITP was used as an intervention to demonstrate venipuncture to the children participating in the study, in order to prepare them for this procedure. This demonstration was performed in the collection room, wards, or outpatient clinic, using a cloth doll and materials such as a needle, syringe, scalpel, saline solution, micropore tape, cotton, and alcohol. The ITP session was performed before venipuncture and after the collection of the first cortisol sample, with the children in the intervention group.

Regarding the participants’ salivary cortisol collection, it was obtained from saliva samples collected with a Salivette, a device consisting of a plastic tube containing a small piece of chewable cotton, which was offered to the child to chew for 3 minutes. The collected material was sent to the laboratory on recyclable ice, as sample stability is guaranteed if refrigerated at 2 to 8°C for 12 hours or frozen at -20°C for up to 6 months.

The cortisol levels used as a normal parameter, according to the circadian rhythm, were those considered in the literature, as expected for all individuals over 1 year of age in the morning period, from 9 a.m. to 12 p.m., ranging from 100 to 670 ng/dL^(6)^.

Regarding the ITP session in the Intervention Group, after collecting a saliva sample for cortisol measurement prior to venipuncture, the researcher demonstrated the puncture on the dummy, using hospital supplies, according to the technique for this procedure and the proposed game.

To do this, ensuring the child’s attention, she inserted the needle into the dummy’s arm after disinfecting it with alcohol, making sensory cues such as, “The needle prick will hurt”, “You can cry, but you can’t pull your arm away”, “When you see the blood coming back from the needle, it’s almost gone”, and concluded the demonstration by attaching the device to the dummy’s arm. Afterwards, the material was offered to the child, inviting them to repeat the procedure with the doll. Following the role-play, which could be repeated as many times as desired, the child was venipunctured, and after 5 to 10 minutes, a second saliva sample was collected. For children in the Control Group, who were not prepared with the ITP, after the venipuncture, a second saliva sample was collected, also 5 to 10 minutes after the stressful stimulus.

### Analysis of results and statistics

Quantitative data analysis was used, comparing salivary cortisol levels of children in the Control Group with those in the Intervention Group, before and after venipuncture, with and without the use of the ITP.

The sample was described according to its baseline characteristics using measures of central tendency, dispersion, and absolute and relative frequencies. All continuous variables were previously assessed for the parametric nature of their distribution^(12)^. The chi-square, Fisher’s exact, Mann-Whitney, and Student’s t-tests were used, with results with a type I error probability of less than 5% (<0.05) considered statistically significant^(12)^.

## RESULTS

### Sociodemographic and clinical data of participants

Table 1 shows that most children were male in both groups, a difference that was statistically significant (p = 0.041); schoolchildren in the Intervention Group and preschoolers in the Control Group, this difference being statistically significant (p = 0.001); with ages ranging from 3 to 11 years and 5 months, with greater variation among the children in the Intervention Group, whose Mean was 7.64 (+ 2.32) and the Median 8, with a statistically significant difference (≤ 0.001); they had chronic disease in both groups, without this difference being statistically significant (p = 0.895); they were successfully punctured on the first attempt, with no statistically significant difference between the groups (p = 0.966); they were hospitalized, both in the Intervention Group and in the Control Group, with no statistically significant difference in their composition (p = 0.237).

**Table 1:** Demographic and clinical data by study group, São Paulo, São Paulo, Brazil, 2023

	Intervention Group	%	Control Group	%	*p* value
GENDER					
Male	16	57.1	20	83.3	0.041a
Female	12	42.8	04	16.7	
AGE GROUP					≤0.001a
< 6 years	7	25.0	17	70.8	
>= 6 years	21	75.0	7	29.2	
DESEASE					0.895a
Acute	11	39.3	9	37.5	
Chronic	17	60.7	15	62.5	
PUNCTURE					0.966b
One	21	75.0	19	79.2	
Two	4	14.3	3	12.5	
Three	2	7.1	1	4.2	
Four	0	0.0	1	4.2	
Five	1	3.6	0	0.0	
SERVICE STATUS					0.237a
Hospitalized	18	64.3	19	79.2	
Outpatient	10	35.7	5	20.8	

*a - Chi-Square Test; b - Fisher's Exact Test.*

### Variation in participants’ cortisol levels

Regarding the normal reference level of cortisol verified before and after venipuncture, it was observed that 14 children (50%) in the Intervention Group and 15 children (62.5%) in the Control Group were within the reference level for cortisol, after the puncture, differences that were not statistically significant (p = 0,474).

### Intervening variables and their influence on participants’ cortisol levels

Table 2 shows that both girls and boys in the Intervention Group showed a decrease in cortisol levels after venipuncture preceded by the intervention with the ITP, both in relation to the Mean and Median, with this difference being greater among boys (0.484), although this result was not statistically significant. (p=0,887b).

**Table 2:** Mean and median of salivary cortisol levels before and after venipuncture of participants in the Intervention Group and Control Group, according to gender, São Paulo, São Paulo, Brazil, 2023

	Intervention Group	Control Group	*p* value
CORTISOL BEFORE	Girls	Girls	
Mean (S.D.)	3.597 (2.834)	9.223 (10.353)	0.358b
Median	3.020	6.212	
CORTISOL AFTER			
Mean (S.D.)	3.301 (2.717)	5.782 (5.458)	0.238b
Median	2.135	5.306	
CORTISOL DIFFERENCE	0.296 (2.353)	3.441 (6.886)	
CORTISOL BEFORE	Boys	Boys	
Mean (S.D.)	5.105 (6.454)	6.219 (8.094)	0.426d
Median	3.325	3.040	
CORTISOL AFTER			
Mean (S.D.)	4.622 (5.013)	5.948 (8.198)	0.464d
Median	2.415	2.850	
CORTISOL DIFFERENCE	0.484 (4.015)	0.271 (4.103)	
*p* value	0.887b	0.219b	

*d - Mann Whitney; b - T-student.*

According to Table 3, among children over 6 years old, there was a reduction in cortisol levels for children in the Intervention Group and an increase for children in the Control Group. Among children under 6 years old, there was a small increase among children in the Intervention Group and a reduction among those in the Control Group. However, the difference in cortisol between children older and younger than 6 years old was not statistically significant, neither in the Intervention Group (p=0.708) nor in the Control Group. (p=0,136).

**Table 3:** Mean and median age-related salivary cortisol levels of participants in the Intervention and Control groups, before and after venipuncture, São Paulo, São Paulo, Brazil, 2023

	Intervention Group	Control Group	*p* value
CORTISOL BEFORE	Children under 6 years old	Children under 6 years old	
Mean	3.107 (2.857)	8.462 (9.399)	0.053d
Median	1.320	3.210	
CORTISOL AFTER			
Mean	3.124 (3.223)	6.750 (8.843)	0.136d
Median	1.820	5.270	
CORTISOL DIFFERENCE	-0.017 (3.938)	1.712 (4.780)	
CORTISOL BEFORE	Higher or = to 6 years	Higher or = to 6 years	
Mean	4.910 (5.756)	2.488 (1.011)	0.542d
Median	3.330	2.410	
CORTISOL AFTER			
Mean	4.366 (4.470)	3.906 (3.541)	0.832d
Median	2.460	2.690	
CORTISOL DIFFERENCE	0.544 (3.227)	-1.418 (3.682)	
*p* value	0.708b	0.136b	

*b - T-student; d - Mann Whitney.*

Regarding the variation in salivary cortisol levels of participants with chronic disease, an increase was identified in the Intervention Group and a reduction in the Control Group. This difference was not statistically significant, although the cortisol result before venipuncture (p=0.052) and the difference in cortisol before and after venipuncture (0.073) presented borderline values, considering a statistical significance of 5%.

Among children with acute disease, comparing the mean and median cortisol levels before and after venipuncture, a decrease was identified in the Intervention Group and an increase in the Control Group, but this difference was not statistically significant. Similarly, the difference in cortisol levels before and after venipuncture was not statistically significant (p=0.159).

In Table 4, comparing the mean and median cortisol levels before and after venipuncture in children who were successfully punctured on the first attempt, a decrease was identified in the Intervention Group and an increase in the Control Group. Although this difference was borderline, it was not statistically significant (P=0.74) and (P=0.51) for the medians of the Intervention and Control groups, respectively.

**Table 4:** Mean and median salivary cortisol levels related to the number of punctures of participants in the Intervention and Control groups, before and after venipuncture, São Paulo, São Paulo, Brazil, 2023

	Intervention Group	Control Group	*p* value
CORTISOL BEFORE with success on the first puncture			
Mean	4.764	7.974	0.074d
Median	3.320	3.390	
CORTISOL AFTER with success on the first puncture			
Mean	4.69	7.028	0.051d
Median	2.93	5.270	
CORTISOL BEFORE with success from the second puncture			
Mean	4.045	1.823	0.227b
Median	3.850	1.400	
CORTISOL AFTER with success from the second puncture			
Mean	4.875	1.850	0.129b
Median	5.490	1.800	
*p* value#	0.214b	0.416d	

*b - T-student; d - Mann Whitney*

Among children who were successfully punctured only on the second attempt, the mean and median cortisol levels increased in the Intervention Group and decreased in the Control Group, which was not statistically significant (P=0.227) and (P=0.129) for the means of the former and the latter, respectively.

## DISCUSSION

The results of this study regarding cortisol levels after venipuncture preceded by the ITP intervention did not demonstrate statistical significance at the 5% level. However, we observed a decrease in cortisol levels in the intervention group after ITP in some participants. It is believed that the sample size may have influenced these results, in addition to the basal cortisol levels of some children in the Intervention Group, who had higher levels before venipuncture compared to children in the Control Group.

Conditions of exacerbated physical and mental stress disrupt cortisol’s inhibitory mechanism on the secretion of adrenocorticotropic hormone (ACTH), increasing its release levels, leading the hypothalamus to secrete more corticotropin-releasing hormone (CRH), with a consequent increase in cortisol release. Therefore, the controlling mechanism, negative feedback, does not operate efficiently under conditions of continuous stress^(13)^.

This may explain why several children in this study, both in the Intervention and Control Groups, had higher cortisol levels than normal even before venipuncture. Seven participants with acute illnesses and six participants with chronic illnesses already had salivary cortisol levels above 670 ng/dL before venipuncture.

However, psychology studies reveal that children and their families use various strategies to cope with the stress generated by illness and hospitalization, also known as coping strategies or ways of coping^(14)^.

Thus, ITP may have been used as a coping strategy by the children in this study, who showed a decrease in cortisol levels after the intervention, temporarily neutralizing ongoing stress, even though the result did not reach statistical significance for the study variables.

The age of the participants in this study may have influenced the decrease in cortisol levels, considering the stress coping strategies that children over 6 years of age have already developed, compared to those under 6 years of age. A randomized clinical study that investigated the effect of play on stress in hospitalized children through urinary cortisol levels resulted in a greater reduction in cortisol levels in school-aged children. It concluded that, at this stage of development, they have more developed abilities to rationalize, understand verbal explanations, express feelings, and tolerate separation from family, demonstrating a greater ability to cope with the stress of hospitalization than younger children^(7)^.

Regarding the impact of venipuncture success on cortisol levels, this study found that 75% of children in the Intervention Group and 79.2% of children in the Control Group were successfully punctured on the first attempt, and salivary cortisol levels decreased after the stressful stimulus, both with and without the use of ITP as an intervention. However, the literature shows that ITP before venipuncture can be used to reduce behavioral changes associated with less adaptation to the procedure and improve children’s acceptance of it^(15)^.

An integrative review on the preparation of children for invasive procedures using TP demonstrated its effectiveness in reducing perioperative anxiety. After its use, children in the experimental group, who received surgical preparation with TP, had lower levels of anxiety compared to those in the control group, who were not prepared for surgery using TP^(16)^.

Another study, also a scoping review, on the impact of TP during childhood hospitalization, although not specifically addressing the use of ITP in preparation for invasive procedures or venipuncture, revealed that play and TP are factors that strengthen bonds between children and their caregivers, both parents and companions, as well as professionals in the multidisciplinary team, bringing a more focused focus on the quality of care^(17)^.

There are many published studies demonstrating that salivary cortisol is a good marker for stress. However, other studies question its effectiveness when compared with other markers, such as salivary chromogranin, also used to measure stress levels. One study found that salivary chromogranin levels increased significantly after venipuncture in children participating in the study, while salivary cortisol levels showed no significant differences^(18,19)^.

### Study limitations

The limitations of this study refer to the inclusion and exclusion factors of participants in the research, since potential participating children were, at many times, under the influence of pain, prolonged fasting, using glucocorticoids, sleep deprived for a long period and with neurological impairment or impairment of the Hypothalamic Pituitary Adrenal (HPA) axis, conditions that limit the possibilities of collecting cortisol.

### Contributions to nursing

Reflecting on the implications for practice and research regarding the aspects revealed by this study, such as the decrease in salivary cortisol levels in children in the group that received venipuncture preparation with ITP, regardless of gender, age, disease, or health care status, we observed what has been published in the scientific literature related to nursing care: that ITP has positive effects on children’s stress^(19,20)^, although this was not statistically proven in this study.

Despite this, behaviors were observed in the children participating in the Intervention Group that showed benefits related to their emotional health. Although manifestations such as crying and irritation were present during the venipuncture, other manifestations were observed afterward, such as smiling, better acceptance of the puncture itself, the medication administered after the puncture, and lively conversations with the researcher and the institution’s health team. Children in the Control Group, on the other hand, remained crying longer after venipuncture, appearing irritated with the researcher and the nursing staff, uncooperative with medication administration, and not open to dialogue with them.

The parents, in turn, were satisfied and grateful for the intervention, even expressing how important the ITP preparation had been for their children, saying it was “something incredible”, a difference in their care. This corroborates the findings of studies conducted with nurses who use TP in pediatric nursing care^(16,21,22)^.

This study also contributed to the observation that physiological markers do not always reveal everything that happens to a child during times of stress and pain, highlighting the need for further research considering other markers of physiological stress, since salivary cortisol has been shown to be volatile and extremely influenced by physical, emotional, and situational factors, and cannot be considered the best marker for measuring child stress.

## CONCLUSIONS

The results obtained allowed us to conclude that the variation in participants’ cortisol levels decreased after venipuncture, with a smaller difference in the Intervention Group than in the Control Group. However, this difference was not statistically significant, demonstrating an asymmetrical distribution of the data.

Considering the median values, cortisol levels decreased in both groups, but to a lesser extent among children who did not receive the ITP intervention.

Regarding the influence of intervening variables on participants’ cortisol levels, regarding gender, both girls and boys in the Intervention Group showed decreased cortisol levels after venipuncture, with the difference being greater among boys (0.484), although this result was not statistically significant (p=0.887). In the Control Group, there was also a reduction in cortisol levels between boys and girls after venipuncture, with the greatest difference observed among girls, although this result was not statistically significant (p=0.219). Regarding age, a reduction in cortisol levels was observed among children over 6 years of age in the Intervention Group and an increase in the Control Group. However, among children under 6 years of age, there was an increase in cortisol levels in the Intervention Group and a reduction in the Control Group. However, the difference was not statistically significant.

Regarding the variable acute or chronic disease, there was an increase in the mean and median cortisol values in the Intervention Group and a reduction in the Control Group. This difference was not statistically significant, although the cortisol result before the puncture (p=0.052) and the difference in cortisol before and after (0.073) showed borderline values, considering a statistical significance of 5% for children with chronic disease.

Among children with acute disease, there was a decrease in the mean and median cortisol values in the Intervention Group and an increase in the Control Group. This difference was not statistically significant, as was the difference in cortisol levels (p=0.159). Regarding the number of puncture attempts, cortisol levels before and after venipuncture in children who were successful on the first attempt were found to decrease in the Intervention Group and increase in the Control Group. This finding reversed among children who were successful only after the second attempt. There was no statistical significance between these values, although borderline values (p=0.051) were found for success on the first puncture.

Thus, it was possible to accept the null hypothesis for this study, despite the fact that it is a new topic in nursing literature. It is worth noting that, although there are numerous studies on TP in the field of nursing and healthcare, their focus is significantly different from that found in the literature.

## Data Availability

The research data are available within the article.
