# Supplementary material for: Influence of therapeutic play on salivary cortisol levels in children undergoing venipuncture
Source: Rev Bras Enferm. 2025 Dec 12;78(Suppl 4):e20240636. doi: 10.1590/0034-7167-2024-0636 (PMC12707757; doi:10.1590/0034-7167-2024-0636)
Supplement: Translation

# Translation: Influência do brinquedo terapêutico nos níveis de cortisol salivar da criança submetida à punção venosa

**DOI:** 10.1590/0034-7167-2024-0636pt

**Authors:** Carina Ceribelli, Fabiane de Amorin Almeida, Circéa Amália Ribeiro

**Affiliations:** I Universidade Cruzeiro do Sul. São Paulo, São Paulo, Brasil; II Faculdade Israelita de Ciências da Saúde Albert Einstein. São Paulo, São Paulo, Brasil; III Universidade Federal de São Paulo. São Paulo, São Paulo, Brasil

**Keywords:** Jogos e Brinquedos, Cateterismo Venoso Periférico, Cortisol, Enfermagem Pediátrica, Hospitalização.

## Abstract

**Objetivos:**

investigar a influência do Brinquedo Terapêutico Instrucional sobre os níveis de cortisol salivar de crianças submetidas à punção venosa periférica.

**Métodos:**

ensaio clínico randômico e controlado, com alocação em dois grupos: Intervenção e Controle, realizado em unidades de internação pediátrica, pronto socorro-infantil e ambulatório de dois hospitais da cidade de São Paulo. A amostra foi composta por 52 crianças, de ambos os sexos, com idade entre 3 e 12 anos, submetidas à punção venosa e aptas a brincar. Os dados foram analisados segundo estatística descritiva e inferencial com nível de significância 5% e intervalo de confiança de 95%.

**Resultados:**

o nível de cortisol diminuiu em ambos os grupos, porém menos entre crianças que não receberam intervenção com o brinquedo; em contrapartida, mantendo-se menor para as que receberam a intervenção e entre aquelas que foram puncionadas com sucesso na primeira tentativa.

**Conclusões:**

Apesar de confirmada a hipótese de nulidade nesta pesquisa, recomenda-se a utilização do Brinquedo Terapêutico para o alívio do estresse.

## INTRODUÇÃO

A motivação para realizar esta pesquisa surgiu de nosso interesse em utilizar e aprofundar o conhecimento sobre tecnologias de cuidado disponíveis para promover a humanização e o alívio do sofrimento da criança doente ou submetida a procedimentos dolorosos e invasivos.

Sabe-se que toda criança precisa brincar para se comunicar, expressar seus sentimentos, suas angústias e para compreender determinadas situações. Brincar é o trabalho da criança, uma atividade essencial a seu bem-estar mental, emocional e social que continua sendo uma necessidade do desenvolvimento, mesmo quando a criança adoece^(1)^. Além disso, brincar é um direito garantido pelo Estatuto da Criança e do Adolescente (ECA) que, em seu Artigo 16 determina à criança o direito à liberdade, que compreende o brincar, praticar esportes e divertir-se^(2)^.

Atividades lúdicas, sejam elas recreacionais ou terapêuticas, podem contribuir para redução do estresse e do sofrimento da criança e, entre elas, destaca-se o Brinquedo Terapêutico (BT), que é tradicionalmente conceituado como um brinquedo estruturado para a criança aliviar a ansiedade causada por experiências atípicas para sua idade, que costumam ser ameaçadoras e requerem mais do que recreação para resolver a ansiedade^(3)^.

Entre os tipos de BT, destacamos o Brinquedo Terapêutico Instrucional (BTI), que deve ser utilizado para a demonstração de procedimentos, fornecendo informações claras, precisas e adequadas ao nível de desenvolvimento da criança, oportunizando que a mesma descarregue sua tensão ao dramatizar as situações vivenciadas e manusear os instrumentos utilizados ou brinquedos que os representem^(4)^.

Estudos apontam que seu uso favorece a redução de comportamentos indicativos de tensão, inclusive durante a realização de procedimentos invasivos no cuidado em saúde, com destaque para a punção venosa^(5)^. Porém, não se identificaram estudos relacionados à influência do BTI sobre um marcador fisiológico de estresse.

Vale ressaltar que é possível medir o nível de estresse na infância por meio da dosagem do hormônio cortisol após um estímulo estressante. O cortisol é um hormônio corticosteróide, produzido pela porção superior da glândula suprarrenal e diretamente envolvido na resposta a estímulos estressantes^(6)^. A dosagem do salivar é recomendada para crianças por ser um método não invasivo, seguro, não doloroso e de fácil realização^(7)^.

Estudo sobre a análise da dor e do cortisol livre em recém-nascidos concluiu que o procedimento de punção venosa repetido intensificou a dor e alterou o cortisol^(8)^. Em outro, realizado com crianças de 5 a 7 anos, hospitalizadas por doença aguda ou crônica, o cortisol salivar matutino e vespertino foi dosado, além de ter sido utilizada uma escala de *cooping* para verificar o estresse, tendo como resultado o aumento do cortisol vespertino quanto menor a idade da criança e que, dentre as estratégias de *cooping* para enfrentamento do estresse decorrente da hospitalização, o brincar foi a mais utilizada pelas crianças^(9)^.

Nesse sentido, observa-se uma lacuna de estudos relacionados à efetividade do uso do BT na diminuição dos níveis de cortisol salivar de crianças expostas aos procedimentos dolorosos e intrusivos, como a punção venosa, o que foi realizado nesta pesquisa.

Assim, neste estudo, em que utilizamos o BTI como estratégia de preparo da criança para a punção venosa, consideramos as seguintes hipóteses de pesquisa a serem investigadas: - Hipótese alternativa (H1): a utilização do BTI no preparo da criança para a punção venosa altera os níveis de cortisol salivar; - Hipótese de nulidade (H0): A utilização do BTI no preparo da criança para a punção venosa não altera os níveis de cortisol salivar.

## OBJETIVOS

Investigar a influência do BTI sobre os níveis de cortisol salivar de crianças submetidas à punção venosa periférica.

## MÉTODOS

### Aspectos éticos

Salienta-se que, atendendo às exigências da Resolução 466/12 do Conselho Nacional de Saúde^(10)^, este estudo foi aprovado pelo comitê de ética em pesquisa da Universidade Federal de São Paulo - UNIFESP via Plataforma Brasil, conforme o parecer anexado a submissão. A pesquisa também foi autorizada pelas instituições onde os dados foram coletados, os pais ou responsáveis assinaram o Termo de Consentimento Livre e Esclarecido, autorizando a participação das crianças na pesquisa e elas mesmas assinaram o Termo de Assentimento.

O estudo foi registrado na plataforma de Registro Brasileiro de Ensaios Clínicos - ReBEC, código de submissão U1111-1287-3284.

### Desenho, período e local do estudo

Trata-se de um ensaio clínico randômico controlado, do tipo paralelo com alocação de 1:1, que foi norteado pela ferramenta CONSORT, sendo realizado o estudo comparativo das dosagens de cortisol salivar com o intuito de verificar se houve redução de seus níveis com o uso do BTI, enquanto indicativo da diminuição do estresse da criança.

Além disso, o período da coleta de dados ocorreu entre setembro de 2018 e outubro de 2022, considerando-se o intervalo de um ano e meio em razão das restrições impostas pela Pandemia de COVID-19.

O estudo foi realizado em três locais na cidade de São Paulo: uma instituição hospitalar pediátrica vinculada à Secretaria de Estado da Saúde do estado, que atende diferentes especialidades pediátricas, sendo referência para tratamento de doenças crônicas de média e alta complexidade; um hospital público terciário de grande porte e alta complexidade, que oferece atendimento ambulatorial, internação, terapia intensiva, cirurgia e urgência e emergência a crianças e adolescentes em várias especialidades; um ambulatório de especialidade, vinculado ao mesmo hospital, que atende pacientes com distúrbios hematológicos, prioritariamente do Sistema Único de Saúde.

### População ou amostra

Ademais, participaram do mesmo 52 crianças atendidas nesses serviços, sendo 24 do Grupo Controle e 28 do Grupo Intervenção, que atenderam aos seguintes critérios de inclusão: terem entre 3 e 12 anos de idade; serem submetidas à punção venosa periférica, durante a internação ou atendimento ambulatorial; estarem em condição clínica favorável que possibilitasse brincar. Os critérios de exclusão foram: estarem em jejum há mais de 12 horas, com dor e fazendo uso de glicocorticóides; privadas de sono há mais de 12 horas; terem comprometimento neurológico ou diagnósticos médicos relacionados ao eixo hipotalâmico-hipofisário-adrenal.

A variável dependente do estudo foi o cortisol salivar, optando-se pela coleta e dosagem por meio de amostras de saliva, que podem ser usadas para monitorar a concentração de uma ampla variedade de drogas, hormônios, anticorpos e outras moléculas^(11)^. Enquanto que a variável independente foi o BTI, ou seja, a ação que poderá afetar a variável dependente, sendo esta, segundo a literatura, o fator a ser manipulado pelo investigador na tentativa de influenciar e assegurar sua relação com a variável dependente, neste caso, o cortisol salivar^(11)^.

### Protocolo do estudo

O BTI foi utilizado como intervenção para demonstrar a punção venosa às crianças participantes do estudo, a fim de prepará-las para esse procedimento, sendo a demonstração realizada na sala de coleta, enfermarias ou ambulatório, utilizando-se de um boneco de pano e de materiais, como agulha, seringa, scalp, soro, fita microporosa, algodão e álcool. A sessão de BTI foi realizada antes da punção venosa e após a coleta da primeira amostra de cortisol, com as crianças do grupo intervenção.

No que se refere à coleta do cortisol salivar dos participantes, o mesmo foi obtido pelas amostras de saliva coletadas com um Salivette, dispositivo composto por tubo plástico, contendo um pequeno pedaço de algodão mastigável, que era ofertado à criança para mastigar durante 3 minutos. O material coletado foi enviado ao laboratório, em gelo reciclável, já que a estabilidade da amostra é garantida se refrigerada de 2 a 8 ºC, por 12 horas, ou congelada a -20 ºC, por até 6 meses.

Os valores de cortisol utilizados como parâmetro de normalidade, de acordo com o ritmo circadiano, foram os considerados pela literatura e conforme esperados para todos os maiores de 1 ano de idade no período da manhã, das 9 às 12 horas, ou seja, variando entre 100 a 670 ng/dL^(6)^.

Quanto à realização da sessão do BTI no Grupo Intervenção, após realizar a coleta da amostra de saliva para dosagem de cortisol anterior à punção venosa, a pesquisadora demonstrava a punção no boneco, utilizando os materiais hospitalares, conforme a técnica desse procedimento e da brincadeira proposta.

Para tanto, certificando-se que a criança estava atenta, introduzia a agulha no braço do boneco, após desinfecção com álcool, fazendo referências sensoriais do tipo: “vai doer a picadinha da agulha”, “você pode chorar, mas não pode puxar o braço”, “quando você vir o sangue voltando na agulha, já estará quase acabando” e finalizava a demonstração fixando o dispositivo no braço do boneco. Após, oferecia o material à criança, convidando-a para repetir o procedimento no boneco e, em seguida à sua dramatização, que podia ser repetida quantas vezes ela desejasse, era realizada a punção venosa da criança e, depois de 5 a 10 minutos, era coletada a segunda amostra da saliva

Em contrapartida, para as crianças do Grupo Controle, as quais não foram preparadas com o BTI, após a realização da punção, também era coletada a segunda amostra de saliva, de 5 a 10 minutos depois do estímulo estressante.

### Análise dos resultados e estatística

A análise quantitativa dos dados foi utilizada através de comparação das dosagens de cortisol salivar das crianças do Grupo Controle com as crianças do Grupo Intervenção, antes e após a punção venosa, com e sem a utilização do BTI.

Logo, a amostra foi descrita conforme suas características basais por meio de medidas de tendência central, dispersão, frequências absolutas e relativas. Todas as variáveis contínuas foram previamente avaliadas quanto à natureza paramétrica de sua distribuição^(12)^. Ainda, foram utilizados os testes estatísticos Qui Quadrado, Teste Exato de Fisher, Mann Whitney e T-student, sendo considerados estatisticamente significativos os resultados com probabilidade de erro tipo I inferior a 5% (< 0,05)^(12)^.

## RESULTADOS

### Dados sóciodemográficos e clínicos dos participantes

De acordo com o Tabela 1, em sua maioria, as crianças eram do sexo masculino nos dois grupos, diferença considerada estatisticamente significante (p=0,041); escolares no Grupo Intervenção e pré-escolares no Grupo Controle, sendo essa diferença também estatisticamente significante (p=0,001); com idade que variou entre 3 e 11 anos e 5 meses, havendo maior variação entre as crianças do Grupo Intervenção, cuja Média foi 7,64(+2,32) e a Mediana 8, com a diferença estatisticamente significante (≤ 0,001); tinham doença crônica em ambos os grupos, sem que essa diferença fosse estatisticamente significante (p=0,895); foram puncionadas com sucesso já na primeira tentativa, sem diferença estatisticamente significante entre os grupos (p = 0,966); encontravam-se hospitalizada, tanto no Grupo Intervenção como no Grupo Controle, similarmente não havendo diferença estatisticamente significante na composição dos mesmos (p=0,237).

**Tabela 1:** Dados demográficos e clínicos, conforme os grupos de estudo, São Paulo, São Paulo, Brasil, 2023

	Grupo Intervenção	%	Grupo Controle	%	Valor de *p*
SEXO					
Masculino	16	57,1	20	83,3	0,041**a**
Feminino	12	42,8	04	16,7	
FAIXA ETÁRIA					≤0,001**a**
< 6 anos	7	25,0	17	70,8	
>= 6 anos	21	75,0	7	29,2	
DOENÇA					0,895**a**
Aguda	11	39,3	9	37,5	
Crônica	17	60,7	15	62,5	
PUNÇÃO					0,966**b**
Uma	21	75,0	19	79,2	
Duas	4	14,3	3	12,5	
Três	2	7,1	1	4,2	
Quatro	0	0,0	1	4,2	
Cinco	1	3,6	0	0,0	
SITUAÇÃO DE ATENDIMENTO					0,237**a**
Hospitalizada	18	64,3	19	79,2	
Ambulatório	10	35,7	5	20,8	

*a - Teste Qui Quadrado; b - Teste Exato de Fisher.*

### Variação dos níveis de cortisol dos participantes

Em relação ao nível de referência de normalidade do cortisol verificado antes e após a punção venosa, observou-se que 14 crianças (50%) do Grupo Intervenção e 15 crianças (62,5%) do Grupo Controle encontravam-se dentro do nível de referência para o cortisol após a punção, diferenças estas que não se mostraram estatisticamente significante (p = 0,474).

### Variáveis intervenientes e sua influência nos níveis de cortisol dos participantes

Observa-se na Tabela 2 que tanto as meninas como os meninos do Grupo Intervenção apresentaram diminuição do nível de cortisol depois da punção venosa antecedida da intervenção com o BTI, tanto em relação à Média como à Mediana, sendo a diferença maior entre os meninos (0,484), embora o resultado não tenha sido estatisticamente significante (p=0,887b).

**Tabela 2:** Média e Mediana relacionadas aos níveis de cortisol salivar, antes e depois da punção venosa dos participantes do Grupo Intervenção e Grupo Controle, em relação ao gênero, São Paulo, São Paulo, Brasil, 2023

	Grupo Intervenção	Grupo Controle	Valor de *p*
CORTISOL ANTES	Meninas	Meninas	
Média (D.P)	3,597 (2,834)	9,223 (10,353)	0,358b
Mediana	3,020	6,212	
CORTISOL DEPOIS			
Média (D.P)	3,301 (2,717)	5,782 (5,458)	0,238b
Mediana	2,135	5,306	
DIFERENÇA CORTISOL	0,296 (2,353)	3,441 (6,886)	
CORTISOL ANTES	Meninos	Meninos	
Média (D.P)	5,105 (6,454)	6,219 (8,094)	0,426d
Mediana	3,325	3,040	
CORTISOL DEPOIS			
Média (D.P)	4,622 (5,013)	5,948 (8,198)	0,464d
Mediana	2,415	2,850	
DIFERENÇA CORTISOL	0,484 (4,015)	0,271 (4,103)	
Valor de *p*	0,887b	0,219b	

*d - Mann Whitney; b - T-student.*

De acordo com a Tabela 3, entre as crianças maiores de 6 anos, verifica-se que houve redução dos níveis de cortisol no Grupo Intervenção, em contraponto ao aumento para as crianças do Grupo Controle. Já entre as menores de 6 anos, houve um pequeno aumento entre as crianças do Grupo Intervenção e uma redução entre as do Grupo Controle. Porém, a diferença do cortisol entre as crianças maiores e menores de 6 anos não foi estatisticamente significante, nem no Grupo Intervenção (p=0,708) nem no Grupo Controle (p=0,136).

**Tabela 3:** Média e Mediana dos níveis de cortisol salivar, relacionadas à idade dos participantes dos grupos Intervenção e Controle, antes e depois da punção venosa, São Paulo, São Paulo, Brasil, 2023

	Grupo Intervenção	Grupo Controle	Valor de *p*
CORTISOL ANTES	Menores de 6 anos	Menores de 6 anos	
Média	3,107 (2,857)	8,462 (9,399)	0,053d
Mediana	1,320	3,210	
CORTISOL DEPOIS			
Média	3,124 (3,223)	6,750 (8,843)	0,136d
Mediana	1,820	5,270	
DIFERENÇA DE CORTISOL	-0,017 (3,938)	1,712 (4,780)	
CORTISOL ANTES	Maior ou = a 6anos	Maior ou = a 6anos	
Média	4,910 (5,756)	2,488 (1,011)	0,542d
Mediana	3,330	2,410	
CORTISOL DEPOIS			
Média	4,366 (4,470)	3,906 (3,541)	0,832d
Mediana	2,460	2,690	
DIFERENÇA DE CORTISOL	0,544 (3,227)	-1,418 (3,682)	
Valor de *p*	0,708b	0,136b	

*b - T-student; d - Mann Whitney.*

Quanto à variação dos níveis de cortisol salivar dos participantes com doença crônica, identificou-se um aumento desse valor no Grupo Intervenção e uma redução no Grupo Controle, diferença que não se mostrou estatisticamente significante, embora o resultado do cortisol antes da punção (p=0,052) e a diferença do cortisol antes e depois (0,073) apresentem valores limítrofes, considerando-se a significância estatística de 5%.

Já entre as crianças com doença aguda, comparando-se a Média e a Mediana do nível de cortisol antes e após a punção venosa, identificou-se uma diminuição desse valor no Grupo Intervenção e um aumento do mesmo no Grupo Controle, mas essa diferença não se mostrou estatisticamente significante. Da mesma forma, a diferença do nível de cortisol antes e depois da punção venosa não apresentou significância estatística (p=0,159).

Na Tabela 4, comparando-se a Média e a Mediana do nível de cortisol, antes e após a punção venosa das crianças que foram puncionadas na primeira tentativa com sucesso, identificou-se uma diminuição desses valores no Grupo Intervenção e um aumento do mesmo no Grupo Controle. Apesar da diferença ter apresentado valores limítrofes, não se mostrou estatisticamente significante, sendo (P=0,74) e (P=0,51) para as Medianas dos grupos Intervenção e Controle, respectivamente.

**Tabela 4:** Média e Mediana dos níveis de cortisol salivar, relacionadas ao número de punções dos participantes dos grupos Intervenção e Controle, antes e depois da punção venosa, São Paulo, São Paulo, Brasil, 2023

	Grupo Intervenção	Grupo Controle	Valor de *p*
CORTISOL ANTES com sucesso na primeira punção			
Média	4,764	7,974	0,074d
Mediana	3,320	3,390	
CORTISOL DEPOIS com sucesso na primeira punção			
Média	4,69	7,028	0,051d
Mediana	2,93	5,270	
CORTISOL ANTES com sucesso a partir de segunda punção			
Média	4,045	1,823	0,227b
Mediana	3,850	1,400	
CORTISOL DEPOIS com sucesso a partir de segunda punção			
Média	4,875	1,850	0,129b
Mediana	5,490	1,800	
Valor de *p*#	0,214b	0,416d	

*b - T-student; d - Mann Whitney*

Já entre as crianças que foram puncionadas com sucesso somente a partir da segunda tentativa, os valores da Média e Mediana do nível de cortisol apresentaram-se aumentados no Grupo Intervenção e diminuídos no Grupo Controle, o que também não apresentou significância estatística (P=0,227) e (P=0,129) para as Médias daquele e deste, respectivamente.

## DISCUSSÃO

Os resultados encontrados nesta pesquisa, em relação ao nível de cortisol após a punção venosa antecedida pela intervenção BTI, não demonstraram significância estatística a 5%. Porém, observamos diminuição de cortisol no grupo intervenção, após o BTI, em alguns participantes. Considera-se que o tamanho da amostra possa ter interferido nesses resultados, além do cortisol basal de algumas crianças do Grupo Intervenção, que apresentaram nível maior antes da punção venosa, em comparação às crianças do Grupo Controle.

Condições de estresse físico e mental exacerbadas atrapalham o mecanismo inibitório do cortisol sobre a secreção do hormônio adrenocorticotrófico (ACTH), aumentando seus níveis de liberação, assim, levando o hipotálamo a secretar mais hormônio liberador da corticotropina (CRH), com consequente aumento também na liberação de cortisol. Portanto, o mecanismo controlador, de feedback negativo, não ocorre de maneira eficiente em condições de estresse contínuo^(13)^.

Consequentemente, isso pode explicar o fato de várias crianças deste estudo, tanto do Grupo Intervenção como do Grupo Controle, apresentarem seus níveis de cortisol mais elevados em relação aos valores de normalidade ainda antes da punção venosa, sendo que sete participantes com doenças aguda e seis participantes com doença crônica já apresentavam seus níveis de cortisol salivar acima de 670 ng/dl, antecedendo a punção venosa.

Entretanto, existem estudos na área de psicologia que revelam que crianças e suas famílias utilizam-se de várias estratégias de enfrentamento de estresse gerado pela doença e hospitalização, também chamadas de *coping* ou *ways of coping* ^(14)^.

Assim, o BTI pode ter sido utilizado como uma estratégia de *coping* pelas crianças deste estudo que apresentaram uma diminuição dos níveis de cortisol após a intervenção, neutralizando temporariamente o estresse contínuo, mesmo que o resultado não tenha alcançado significância estatística para as variáveis da pesquisa.

Ademais, a idade dos participantes desta pesquisa pode ter influenciado a diminuição ou não dos níveis de cortisol, considerando as estratégias de enfrentamento de estresse (*coping*) que crianças maiores de 6 anos já desenvolveram, isso se comparadas com as menores de 6 anos. Ressalta-se que estudo clínico randomizado, que investigou por meio dos níveis de cortisol urinário o efeito da brincadeira sobre o estresse da criança hospitalizada, teve como resultado uma maior redução do cortisol das crianças em idade escolar, concluindo que, nessa fase do desenvolvimento, elas têm habilidades de racionalizar, entender explicações verbais, expressar sentimentos e tolerar a separação da família mais desenvolvidas, logo, mostrando maior capacidade de lidar com o estresse da hospitalização do que as crianças menores^(7)^.

Em relação à interferência do sucesso da punção venosa nos níveis de cortisol, nesta pesquisa verificou-se que 75% das crianças do Grupo Intervenção e 79,2% das crianças do Grupo Controle foram puncionadas já com sucesso na primeira tentativa e, consequentemente, os níveis de cortisol salivar diminuíram, após o estímulo estressante com e sem a utilização do BTI como intervenção. Entretanto, a literatura mostra que o BTI antecedendo a punção venosa pode ser utilizado para reduzir alterações comportamentais de menor adaptação ao procedimento e melhorar a aceitação do mesmo pelas crianças^(15)^.

Uma revisão integrativa sobre o preparo de crianças para procedimentos invasivos com o uso do BT apontou a efetividade do mesmo na redução da ansiedade perioperatória, mostrando que, após seu uso, as crianças do grupo experimental - que receberam preparo para cirurgia com BT - apresentavam níveis inferiores de ansiedade quando comparadas com as do grupo controle, que não foram preparadas para a cirurgia utilizando-se o BT^(16)^.

Outro estudo, também do tipo revisão de escopo, sobre o impacto do BT durante a hospitalização na infância, embora não específico sobre o uso do BTI no preparo para procedimentos invasivos ou a punção venosa, revelou que o lúdico e o BT são fatores de estreitamento de laços entre as crianças e seus cuidadores, tanto os pais e acompanhantes quanto os profissionais da equipe multidisciplinar, trazendo um olhar mais voltado à qualidade da assistência^(17)^.

Além do que, há muitos trabalhos publicados demonstrando ser o cortisol salivar um bom marcador para o estresse. Porém, outros estudos questionam sua eficácia quando comparado com outros marcadores, como a cromogranina salivar, também usada para medir os níveis de estresse. E, ainda, uma pesquisa observou que os níveis de cromogranina salivar aumentaram significativamente após a punção venosa nas crianças participantes do estudo, enquanto os níveis de cortisol salivar não demonstraram diferenças significativas^(18,19)^.

### Limitações do estudo

As limitações deste estudo referem-se aos fatores de inclusão e exclusão dos participantes na pesquisa, já que crianças potenciais participantes encontravam-se, em muitos momentos, sob a influência de dor, jejum prolongado, em uso de glicocorticoides, privados de sono por um longo período e com comprometimento neurológico ou do eixo Hipotalâmico Hipofizário Adrenal (HHA), condições limitadoras das possibilidades da coleta de cortisol.

### Contribuições para a enfermagem:

Refletindo sobre as implicações para prática e a pesquisa, quanto aos aspectos revelados por este estudo, como exemplo a diminuição do cortisol salivar de crianças do grupo que recebeu o preparo para a punção venosa com o BTI, independente do sexo, idade, doença ou condição de atendimento no serviço de saúde, foi observado resultados similares com o que vem sendo publicado na literatura científica relacionada à assistência de enfermagem: que o BTI tem efeitos positivos sobre o estresse da criança^(19,20)^, embora isso não tenha sido comprovado estatisticamente neste estudo.

Apesar disso, observou-se comportamentos nas crianças participantes do Grupo Intervenção, que mostraram benefícios relacionados à saúde emocional das mesmas, pois embora manifestações como choro e irritação se fizessem presentes no momento da punção venosa, após a mesma houveram outras manifestações como sorrisos, melhor aceitação da punção propriamente dita, da medicação administrada após a punção, bem como conversas animadas com a pesquisadora e com a equipe de saúde da instituição. Já as crianças do Grupo Controle permaneceram chorando por mais tempo após a punção venosa, mostrando-se irritadas com a pesquisadora e com a equipe de enfermagem, não colaborativas à administração do medicamento e não dando abertura ao diálogo.

Por sua vez, os pais mostravam-se satisfeitos e agradecidos pela intervenção, chegando a verbalizar o quanto o preparo com o BTI havia sido importante para seus filhos, sendo “algo incrível”, um diferencial no cuidado dos mesmos, o que corrobora com os achados em estudos realizados com enfermeiros que utilizam o BT na assistência de enfermagem pediátrica^(16,21,22)^.

Em suma, este estudo contribuiu também para observar que marcadores fisiológicos nem sempre revelam tudo o que acontece com a criança em um momento de estresse e dor, ressaltando a necessidade de serem realizadas novas pesquisas considerando outros marcadores de estresse fisiológico, uma vez que o cortisol salivar se mostrou volátil e extremamente influenciado por fatores físicos, emocionais e situacionais, não podendo ser considerado o melhor marcador para medir o estresse da criança.

## CONCLUSÕES

Os resultados obtidos permitiram concluir que a variação dos níveis de cortisol dos participantes apresentou diminuição dos valores, após a punção venosa, sendo sua diferença menor no Grupo Intervenção do que no Grupo Controle; entretanto, essa diferença não foi estatisticamente significante, verificando-se uma distribuição assimétrica dos dados. Sendo que, considerando-se o valor das medianas, o nível de cortisol diminuiu em ambos os grupos, porém em menor quantidade entre as crianças que não receberam a intervenção com o BTI.

Já em relação à influência das variáveis intervenientes nos níveis de cortisol dos participantes quanto ao gênero, tanto as meninas quanto os meninos do Grupo Intervenção apresentaram diminuição do nível de cortisol depois da punção venosa, sendo essa diferença maior entre os meninos (0,484), ainda que o resultado não tenha sido estatisticamente significante (p=0,887). Em contrapartida, no Grupo Controle, que também houve redução dos níveis de cortisol entre meninos e meninas após a punção venosa, sendo a maior diferença observada entre as meninas e não sendo o resultado estatisticamente significante (p=0,219).

Quanto à variável idade, observou-se redução dos níveis de cortisol entre as crianças maiores de 6 anos no Grupo Intervenção e aumento no Grupo Controle, mas entre as crianças menores de 6 anos houve aumento dos níveis de cortisol no Grupo Intervenção e redução no Grupo Controle, porém, a diferença não foi estatisticamente significante.

No que diz respeito à variável doença aguda ou crônica, houve aumento dos valores da Média e Mediana do cortisol no Grupo Intervenção e uma redução no Grupo Controle, diferença que similarmente as demais não se mostrou estatisticamente significante, embora o resultado do cortisol antes da punção (p=0,052) e a diferença do cortisol antes e depois (0,073) tenham apresentado valores limítrofes, considerando-se a significância estatística de 5% para as crianças com doença crônica.

Já entre as crianças com doença aguda, houve a diminuição dos valores da Média e Mediana do cortisol no Grupo Intervenção e aumento no Grupo Controle, diferença que não se mostrou estatisticamente significante, assim como no que se refere a diferença do nível de cortisol (p=0,159).

Ademais, relacionado ao número de tentativas de punção, o nível de cortisol antes e após a punção venosa das crianças que tiveram sucesso na primeira tentativa, identificou-se a diminuição desse valor no Grupo Intervenção e o aumento no Grupo Controle, ocorrendo uma inversão deste achado entre as crianças com sucesso somente a partir da segunda tentativa. Não houve significância estatística entre esses valores, embora tenha-se verificado valores limítrofes (p=0,051) relacionados ao sucesso na primeira punção.

Por fim, foi possível aceitar a hipótese de nulidade nesta pesquisa, apesar de tratar-se de uma temática inédita na literatura de enfermagem. Destaca-se que, embora existam inúmeros estudos sobre o BT no campo da enfermagem e da saúde, seu enfoque é significativamente diferente daqueles observados nos demais trabalhos encontrados na literatura.

## Data Availability

Os dados da pesquisa estão disponíveis no corpo do artigo.
